# Effects of *Bacillus amyloliquefaciens* Instead of Antibiotics on Growth Performance, Intestinal Health, and Intestinal Microbiota of Broilers

**DOI:** 10.3389/fvets.2021.679368

**Published:** 2021-06-04

**Authors:** Baikui Wang, Yuanhao Zhou, Li Tang, Zihan Zeng, Li Gong, Yanping Wu, Wei-Fen Li

**Affiliations:** ^1^Key Laboratory of Animal Molecular Nutrition of Education of Ministry, National Engineering Laboratory of Biological Feed Safety and Pollution Prevention and Control, Key Laboratory of Animal Feed and Nutrition of Zhejiang Province, Institute of Animal Nutrition and Feed Sciences, College of Animal Sciences, Zhejiang University, Hangzhou, China; ^2^School of Life Science and Engineering, Foshan University, Foshan, China

**Keywords:** antioxidant capacity, digestive function, immune function, intestinal epithelial barrier, intestinal microbiota, *Bacillus amyloliquefaciens*

## Abstract

The aim of this study was to evaluate the dietary effects of *Bacillus amyloliquefaciens* SC06 (SC06) instead of antibiotics on the growth performance, intestinal health, and intestinal microbiota of broilers. A total of 360 30-day-old Lingnan yellow broilers were randomly allocated into two groups with six replicates per group (30 birds per replicate). The broilers were fed either a non-supplemented diet or a diet supplemented with 10^8^ colony-forming units lyophilized SC06 per kilogram feed for 30 days. Results showed that SC06 supplementation had no effect on the growth performance compared with that of the control group. SC06 treatment significantly (*P* <0.05) increased the total antioxidant capacity (T-AOC), total superoxide dismutase (T-SOD) activity in the liver, and the activities of trypsin, α-amylase (AMS), and Na^+^K^+^-ATPase in the ileum, whereas it decreased (*P* < 0.05) lipase, gamma glutamyl transpeptidase (γ-GT), and maltase activities in the ileum. Meanwhile, SC06 treatment also improved the immune function indicated by the significantly (*P* < 0.05) increased anti-inflammatory cytokine [interleukin (IL)-10] level and the decreased (*P* < 0.05) pro-inflammatory cytokine [IL-6 and tumor necrosis factor (TNF)-α] levels in the ileum. Furthermore, we also found that SC06 enhanced the intestinal epithelial intercellular integrity (tight junction and adhesion belt) in the ileum. Microbial analysis showed that SC06 mainly increased the alpha diversity indices in the jejunum, ileum, and cecum. SC06 treatment also significantly (*P* < 0.05) increased the abundances of *Bacteroidetes, Bacteroidales, Bacteroides, Fusobacteria, Clostridiaceae*, and *Veillonellaceae* in the cecum and simultaneously decreased the abundances of *Planococcaceae* in the duodenum, *Microbacteriaceae* in the jejunum, and *Lachnospiraceae, [Ruminococcus]* and *Ruminococcus* in cecum. In conclusion, these results suggested that *B. amyloliquefaciens* instead of antibiotics showed a potential beneficial effect on the intestinal health of broilers.

## Introduction

Over the past decades, the antimicrobial growth promoters (AGPs) have enjoyed great popularity in improving growth performance, reducing pathogenic bacterial colonization, and preventing gut disease in poultry (1, 2). However, with increasing public concerns about antibiotic-resistant bacteria and antibiotic-residual animal products, AGPs have been widely prohibited in animal husbandry in many countries (3–5). Subsequently, with the strict prohibition of the in-feed AGPs, gastrointestinal pathogenic infectious diseases in food animal production and zoonotic pathogen contamination in animal products seriously threaten the health of animals and humans (6, 7). Therefore, it is very important to explore the proper alternatives to antibiotics, such as direct-fed microbes (DFMs), prebiotics, antimicrobial peptides, plant extracts, immune activators, and organic acidifiers.

As live microorganisms, probiotics do not leave residues in food animal products and have been widely used in animal husbandry to improve growth performance and overall health and can be regarded as a potential substitute for AGPs (8–10). It is reported that probiotics, such as *Bacillus* spp., *Lactobacillus, Bifidobacterium*, and *yeasts*, play an important role in regulating birds' intestinal microbiota, inhibiting pathogens' growth, and modulating gastrointestinal immune responses (11–13). Our previous studies found that *B. amyloliquefaciens* SC06 (SC06) supplementation instead of antibiotics could significantly improve the growth performance of piglets *via* increasing antioxidant capacity and intestinal autophagy, suggesting that it could be used as a potential alternative to antibiotics in animal husbandry (9, 14). Moreover, SC06 could effectively protect intestinal porcine epithelial cell 1 (IPEC-1) from oxidative stress by regulating reactive oxygen species (ROS) production, activating the Nrf2/Keap1 signaling pathway, and promoting the elimination of *Escherichia coli* in murine macrophage RAW264.7 cells by activating autophagy (15). However, the effects of *B. amyloliquefaciens* SC06 on broilers' performance and healthy status remain unclear. Therefore, the objective of this study was to evaluate the effects of *B. amyloliquefaciens* SC06 as a potential antibiotic substitute on the growth performance, intestinal health, and intestinal microbiota of broilers.

## Materials and Methods

### Bacterial Preparation

*Bacillus amyloliquefaciens* SC06 was isolated from soil and deposited in the China Center for Type Culture Collection (CCTCC No. M 2012280). Bacteria were cultured in Luria–Bertani broth overnight at 37°C and then harvested by centrifugation at 5,000 rpm for 15 min. After washing twice with sterile phosphate-buffered saline (PBS; pH = 7.4), SC06 was resuspended in PBS and the concentration was constantly checked by the spreading plate method (16).

### Birds, Diet, and Management

Three hundred sixty 30-day-old Lingnan yellow broilers were randomly allocated into two groups with six pens per group (30 birds per pen, stocking density 0.38 m^2^/bird). Broilers in the control group were fed with the basal diet containing colistin sulfate (10 g/ton) and zinc bacitracin (40 g/ton). Broilers in the SC06 group were fed with the same basal diet (without zinc bacitracin) supplemented with *B. amyloliquefaciens* SC06 (1 × 10^8^ cfu/kg feed). The broiler experiment lasted for 30 days. All birds were allowed *ad libitum* to access water and diets. Feed consumption was recorded every day, and body weight was recorded on days 30 and 60. The composition of the basic diet (Table 1) was formulated to meet nutrient requirements of Chinese yellow-feathered broilers (17). Daylight was eliminated during this study, and 18-h lighting was provided from incandescent bulbs. Mortality was checked daily, and dead birds were weighed to adjust estimates of body weight gain, feed intake, and feed conversion ratio.

**Table 1:** Composition and nutrient levels of the basal diet (% as fed basis).

**Ingredients**	**Content (%)**	**Nutrient levels^b^**	**Content (%)**
Corn	64.00	ME (Mcal/kg)	3.05
Soybean meal	23.25	Lysine	0.98
Soybean oil	3.65	Methionine	0.36
Feather meal	3.50	Methionine + cysteine	0.73
Limestone	1.17	Threonine	0.75
Dicalcium phosphate	1.65	Tryptophan	0.21
Methionine	0.09	Isoleucine	0.77
Sodium chloride	0.30	CP	18.95
Zeolite powder	1.39	Calcium	0.90
Premix^a^	1.00	Non-phytate phosphorus	0.40

a *Supplied per kilogram of diet: Vitamin A, 9,375 IU; Vitamin D_3_, 2,500 IU; Vitamin E, 80.00 mg; Vitamin K, 2.94 mg; Vitamin B1, 2.50 mg; Vitamin B_2_, 6.25 mg; pantothenic acid, 30.30 mg; pyridoxine, 9.09 mg; biotin, 22.50 mg; folic acid, 1.67 mg; Vitamin B_12_, 3.00 mg; ZnSO_4_·H_2_O, 180.93 mg; CuSO_4_·H_2_O, 33.18 mg; FeSO_4_·H_2_O, 247.75 mg; MnSO_4_·H_2_O, 248.45 mg; Ca(IO_3_)_2_, 85.80 mg; Na_2_SeO_3_, 37.60 mg*.

b *Calculated nutrient levels*.

### Sample Collection

At the 60th day of the trial, birds were deprived of feed for 4 h (05:00~09:00 a.m.) but not water. Six birds from each group were then chosen randomly and weighed. Then, the birds were electrically stunned, exsanguinated, and scalded to enable to collect tissues. The mucosa of ileum segments was gently scraped, along with the liver, snap frozen in liquid nitrogen, and stored at −80°C for further experiments.

### Antioxidant Capacity Assay

The liver samples were homogenized with ice-cold sterile saline solution (1:9, w/v) and centrifuged at 5,000 rpm for 25 min at 4°C. Then, the collected supernatant was stored at −80°C for further enzyme activity assays. Briefly, after thawing the homogenates, adjusting to room temperature, the capacity of total antioxidant capacity (T-AOC), the activities of total superoxide dismutase (T-SOD), glutathione peroxidase (GSH-Px), xanthine oxidase (XOD), and superanion oxide (O^2−^) and content of glutathione (GSH) were analyzed by a SpectraMax M5 (Molecular Devices, USA) using assay kits (Nanjing Jiancheng Bioengineering Institute, Nanjing, China) according to the instructions of the manufacturer.

### Digestive Enzyme Activity Assay

The ileum mucosa samples were homogenized with ice-cold sterile saline solution (1:9, w/v) and centrifuged at 5,000 rpm for 25 min at 4°C. Then, the collected supernatant was stored at −80°C for enzyme assays. Briefly, after thawing the homogenates, adjusting to room temperature, the activities of trypsin, lipase, α-amylase (AMS), gamma glutamyl transpeptidase (γ-GT), Na^+^K^+^ ATPase, sucrase, and maltase were analyzed by a SpectraMax M5 (Molecular Devices, USA) using assay kits (Nanjing Jiancheng Bioengineering Institute, Nanjing, China) according to the instructions of the manufacturer.

### ELISA

The cytokine levels of interleukin (IL)-6, tumor necrosis factor (TNF)-α, IL-10, transforming growth factor (TGF)-β, interferon (IFN)-γ, IFN-α, and secretory immunoglobulin A (sIgA) in the ileum mucosa homogenates were determined by enzyme-linked immunosorbent assay (ELISA) kits (Bio-function Technology Co., Ltd., Beijing, China) according to the instructions.

### Transmission Electron Microscopy

After fixing in 2.5% buffered glutaraldehyde, the ileum tissues were washed three times in cold 0.1 M phosphate buffer at every 15-min interval. The tissues were post-fixed in cold 0.1% buffered osmium tetroxide (OsO_4_) for 2 h and washed again in phosphate buffer. After rapidly dehydrating in an ascending serial ethanol solution (30%, 50%, 70%, 95%, and 100%), the tissues were then transferred to a 1:1 mixture of propylene oxide and epoxy araldite. After embedding, ultrathin sections (60–100 nm) were cut with an LKB Nova ultra-microtome (Leica Microsystems, Buffalo Grove, IL) and stained with uranyl acetate. Electron micrographs of intestinal mucosal cells and microvilli were captured by the transmission electron microscope (JEOL, Tokyo, Japan) operating at 80 kV.

### Microbial Analysis

The DNA Isolation Kit (Tiangen, Beijing, China) was used for bacterial genomic DNA extraction from the contents of the duodenum, jejunum, ileum, and cecum, and the quality of the extracted DNA was checked by agarose gel electrophoresis and spectrophotometric analysis. All the genomic DNA samples were stored at −80°C for further experiments. Here, 16s rRNA PCR amplification and 454 pyrosequencing were performed according to a previous study (18). Sequences obtained through 454 pyrosequencing were then filtered by QIIME software (QIIME version 1.9.1) with default parameters. The operational taxonomic unit (OTU) clustering pipeline UPARSE was used to select OTU at 97% similarity. Alpha diversity and beta diversity between the samples were also analyzed by QIIME software. Beta diversity was displayed by principal coordinates analysis (PCoA) using the “ape” package of R software. Permutational multivariate analysis of variance (PERMANOVA) was calculated to determine significant differences in microbial community (based on the Bray–Curtis distance matrices). The linear discriminant analysis (LDA) effect size (LEfSe) analysis was performed online (https://huttenhower.sph.harvard.edu/galaxy/) to find out the highly dimensional intestinal microbes and characterize the differences between the two groups. The biomarkers were then analyzed and visualized by statistical analysis of taxonomic and functional profiles (STAMP) software with a two-sided Welch's *t*-test (19).

Co-occurrence networks of the microbial communities in the different intestinal segments between the two groups were built based on significant correlations [Spearman's R > 0.6 and false discovery rate (FDR)-adjusted *P* < 0.05] (20) and were visualized by Gephi software (https://gephi.org/). The complex patterns of the interrelationships were described by the topological properties of co-occurrence network calculated by Gephi software.

### Statistical Analysis

The rest of the data were analyzed by two-tailed Student's *t*-test using SPSS 20.0 (SPSS Inc., Chicago, IL, USA), and results were expressed as mean ± standard error of the mean (SEM).

## Results

### Growth Performance

As shown in Table 2, compared with the control group, SC06 treatment had no (*P* > 0.05) effect on the growth performance, including the final body weight, the average daily feed intake, the average daily gain, and the ratio of feed conversion, of broilers.

**Table 2:** Effects of SC06 on growth performance of broilers.

**Items**	**Control**	**SC06**
Initial body weight (kg/bird)	1.68 ± 0.004	1.73 ± 0.018
Final body weight (kg/bird)	2.11 ± 0.013	2.17 ± 0.022
Average daily feed intake (g/day/bird)	39.42 ± 0.514	40.35 ± 0.494
Average daily gain (g/day/bird)	14.42 ± 0.555	14.61 ± 0.233
Feed conversion ratio	2.71 ± 0.022	2.76 ± 0.031

*Values are mean ± standard error of the mean (SEM) with six pens*.

### Antioxidant Capacity in Liver

As shown in Table 3, SC06 treatment significantly (*P* < 0.05) increased the T-AOC capacity and T-SOD activity of liver but had no (*P* > 0.05) effect on the content of GSH and O^2−^ and the activities of GSH-Px and XOD.

**Table 3:** Effects of SC06 on liver antioxidant parameters of broilers.

**Items**	**Control**	**SC06**
T-AOC (U/mgprot)	6.41 ± 0.46^b^	8.41 ± 0.57^a^
T-SOD (U/mgprot)	25.55 ± 1.02^b^	29.47 ± 0.54^a^
GSH-Px (U/mgprot)	248.54 ± 21.14	242.11 ± 22.59
GSH (mg/gprot)	345.34 ± 60.88	333.08 ± 21.35
O^2−^ (U/gprot)	238.29 ± 11.82	232.40 ± 8.98
XOD (U/gprot)	200.61 ± 17.10	195.72 ± 11.30

*Values are mean ± standard error of the mean (SEM) with six samples. Different letters indicate significant differences (P < 0.05) among the groups. T-AOC, total antioxidation capacity; T-SOD, total superoxide dismutase; GSH-Px, glutathione peroxidase; GSH, glutathione; O^2−^, superanion oxide; XOD, xanthione oxidase*.

### Digestive Enzyme Activities in the Ileum

Compared with the control group, SC06 treatment significantly (*P* < 0.05) increased the activities of trypsin, AMS, and Na^+^K^+^-ATPase in the ileum, whereas it significantly (*P* < 0.05) decreased the activities of lipase, γ-GT, and maltase (Table 4).

**Table 4:** Effects of SC06 on digestive enzyme activities in the ileum of broilers.

**Items**	**Control**	**SC06**
Trypsin (U/mgprot)	825.98 ± 29.47^b^	1,563.39 ± 45.76^a^
Lipase (U/gprot)	49.24 ± 0.43^a^	31.57 ± 0.85^b^
AMS (U/mgprot)	0.43 ± 0.04^b^	1.03 ± 0.16^a^
γ-GT (U/gprot)	78.98 ± 4.80^a^	48.96 ± 3.76^b^
Na^+^K^+^-ATPase (U/mgprot)	8.28 ± 0.22^b^	14.9 ± 0.33^a^
Sucrase (U/mgprot)	162.05 ± 6.95	177.61 ± 2.89
Maltase (U/mgprot)	637.5 ± 21.49^a^	439.58 ± 11.30^b^

*Values are mean ± standard error of the mean (SEM) with six samples. Different letters indicate significant differences (P < 0.05) among the groups. AMS, α-amylase; γ-GT, gamma glutamyl transpeptidase*.

### Cytokine Levels in the Ileum

SC06 treatment significantly (*P* < 0.05) increased IL-10 level and decreased the concentrations of IL-6 and TNF-α in the ileum, whereas it had no effect (*P* > 0.05) on the levels of TGF-β, IFN-γ, IFN-α, and sIgA (Table 5).

**Table 5:** Effects of SC06 on cytokine levels in the ileum of broilers.

**Items**	**Control**	**SC06**
IL-6 (ng/g tissue)	589.50 ± 0.38^a^	457.31 ± 17.76^b^
TNF-α (ng/g tissue)	1,105.18 ± 23.75^a^	991.20 ± 16.66^b^
IFN-γ (pg/g tissue)	1,486.75 ± 30.25	1,490.03 ± 71.26
IFN-α (ng/g tissue)	1,646.57 ± 12.47	1,750.30 ± 100.97
IL-10 (ng/g tissue)	19.56 ± 1.16^b^	28.23 ± 0.41^a^
TGF-β (pg/g tissue)	1,921.17 ± 23.15	1,788.13 ± 71.66
sIgA (μg/g tissue)	67.08 ± 0.74	72.4 ± 3.29

*Values are mean ± standard error of the mean (SEM) with six samples. Different letters indicate significant differences (P < 0.05) among the groups. IL-6, interleukin-6; TNF-α, tumor necrosis factor α; TGF-β, transforming growth factor-β; IFN-γ, interferon-γ; sIgA, secretory immunoglobulin A*.

### Transmission Electron Micrograph

**Figure 1:**
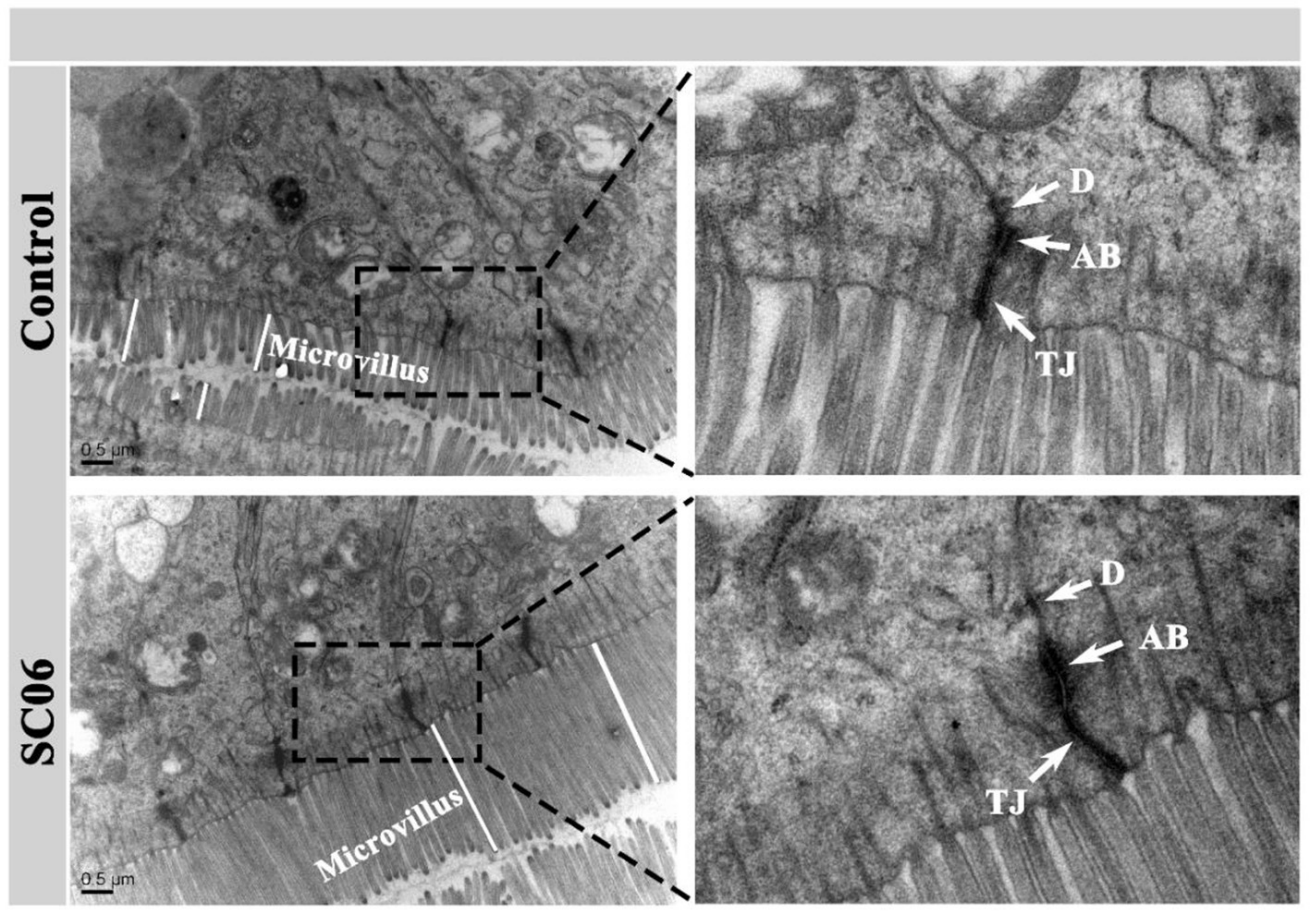
Transmission electron micrographs (TEMs) of the ileal microvilli in broilers. TJ, tight junction; AB, adhesion belt; D, desmosome.

TEM results showed that the ileum of the broilers fed with SC06 showed ordered arrangement, higher microvillus, and longer tight junction (TJ) and adhesion belt (AB) compared with broilers fed with antibiotics (Figure 1).

### Intestinal Microbiota Analysis

**Figure 2:**
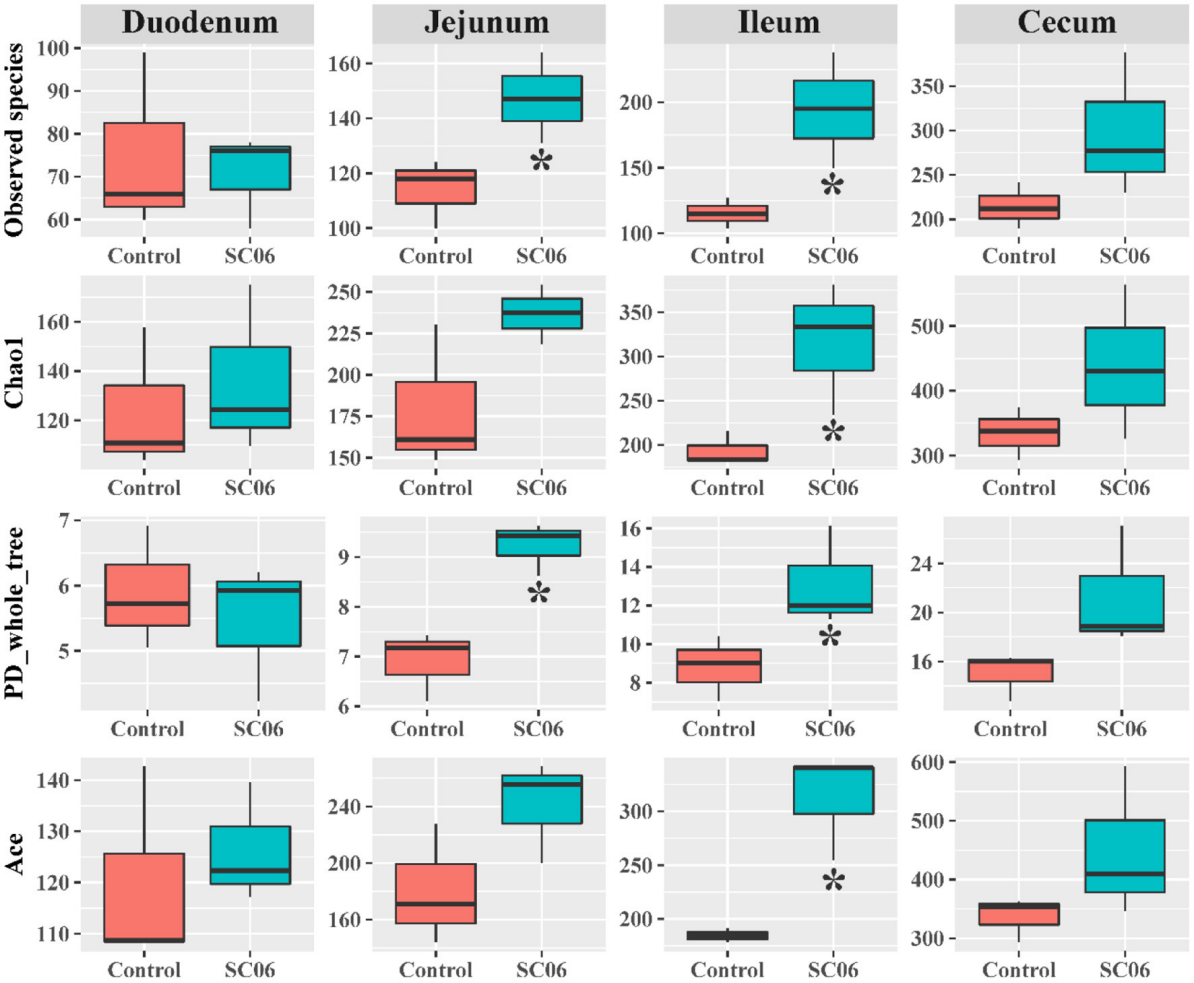
Alpha diversity index analysis of the intestinal microbiota between the two groups (*n* = 3 birds/group). *P < 0.05.

**Figure 3:**
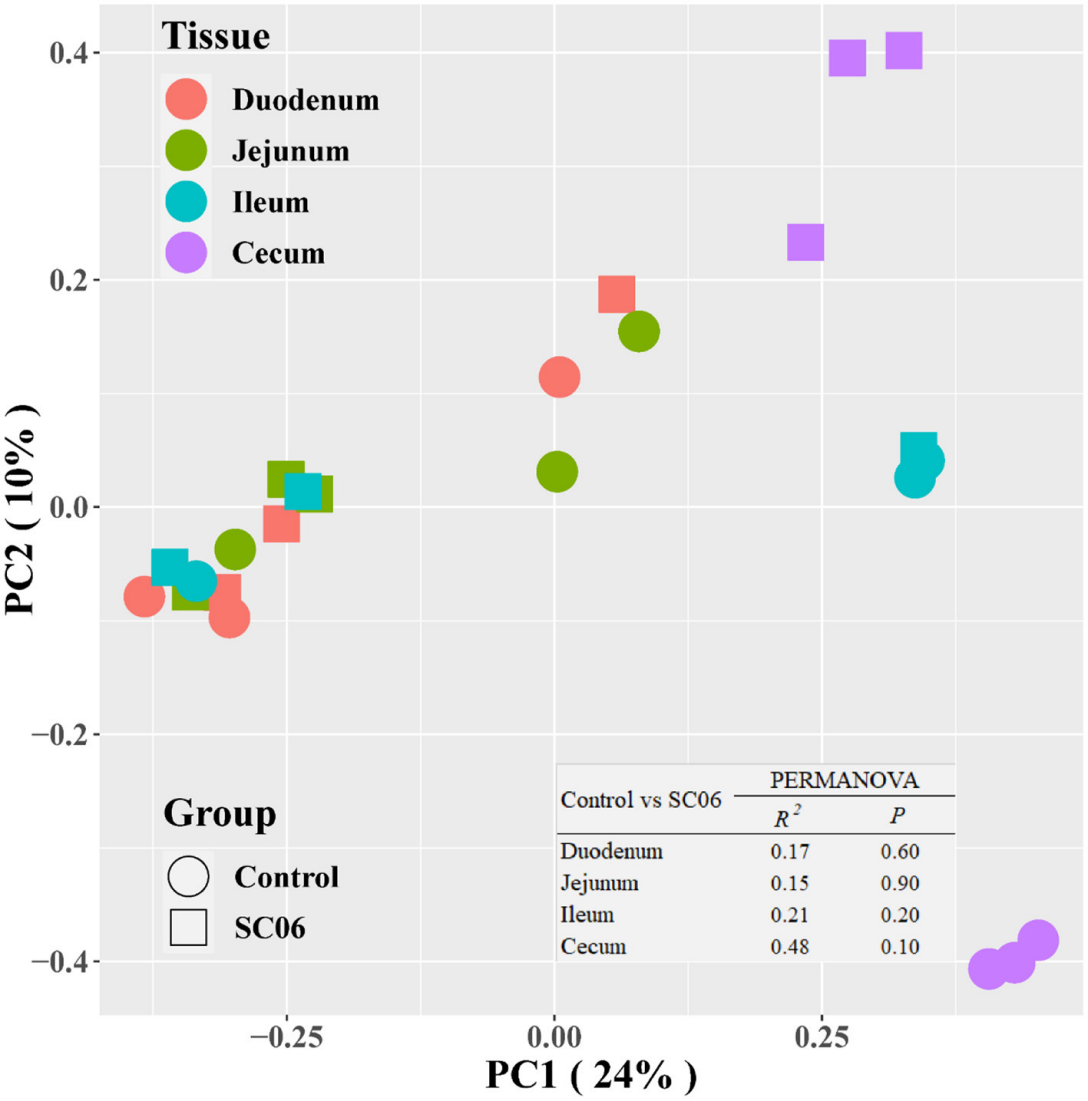
Microbial community analyzed by principal coordinates analysis (PCoA) based on Bray–Curtis distance. PERMANOVA, permutational multivariate analysis of variance (*n* = 3 birds/group).

The intestinal microbiota plays an important role in the overall health of the broilers. We found that SC06 treatment significantly (*P* < 0.05) increased the alpha diversity indices (including Observed species, Chao1, PD_whole_tree, and Ace) of ileal microbiota and the indices of Observed species and PD_whole_tree in the jejunum (Figure 2). SC06 treatment also increased (*P* > 0.05) the alpha diversity indices of cecal and duodenal microbiota. PCoA of microbial communities based on Bray–Curtis distance revealed that bacterial communities from the ileum and cecum formed distinct clusters but had no significant differences between the two groups, which was further confirmed by PERMANOVA analysis (ileum: *R*^2^ = 0.21, *P* = 0.20; cecum: *R*^2^ = 0.48, *P* = 0.10) (Figure 3).

**Figure 4:**
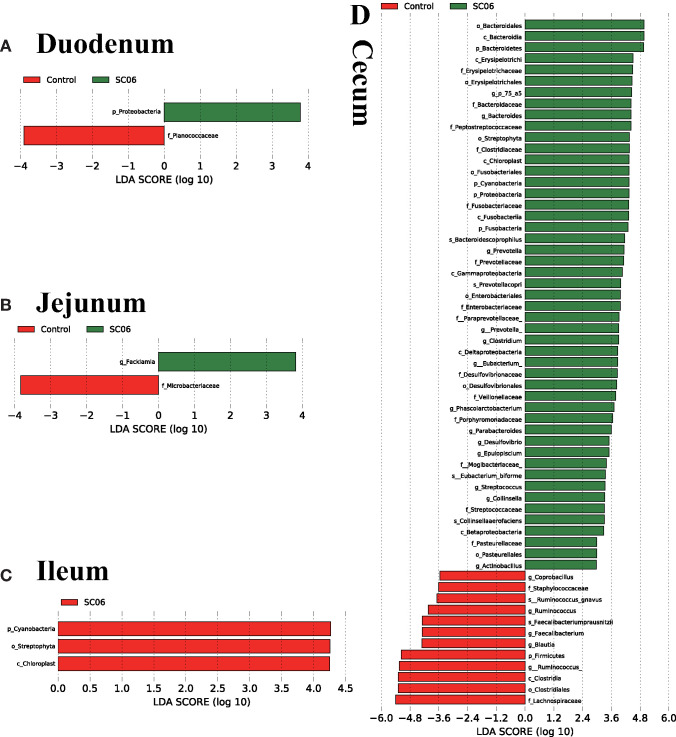
Linear discriminant analysis (LDA) effect size (LEfSe) analysis (*P* < 0.05, LDA >2.0) showing the biomarker taxa (*n* = 3 birds/group). The prefixes “p,” “c,” “o,” “f,” “g,” and “s” represent the annotated levels of phylum, class, order, family, genus, and species. **(A)** duodenum, **(B)** jejunum, **(C)** ileum, **(D)** cecum.

**Figure 5:**
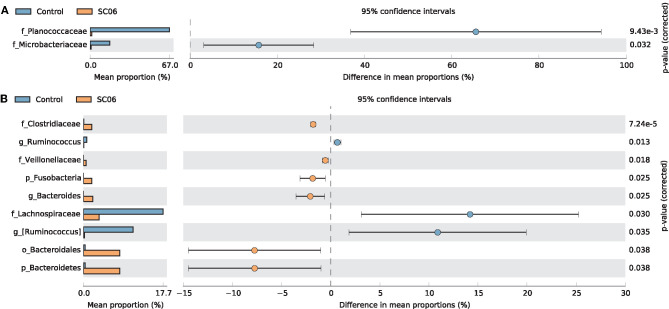
Comparison of the intestinal microbiota between the two groups by statistical analysis of taxonomic and functional profiles (STAMP). **(A)** Differences of *Planococcaceae* in the duodenum and *Microbacteriaceae* in the jejunum. **(B)** Differences of intestinal microbiota in the cecum.

LEfSe analysis showed that 68 biomarkers were identified with LDA scores >2, of which 61 biomarkers were identified in cecal microbiota (Figure 4). The SC06 group was enriched with *Proteobacteria* in the duodenum and *Facklamia* in the jejunum, while the control group was enriched with *Planococcaceae* in the duodenum, *Microbacteriaceae* in the jejunum, and *Cyanobacteria, Chloroplast*, and *Streptophyta* in the ileum. In the cecum, SC06 group was enriched with 49 biomarkers, most of which belonged to *Firmicutes, Proteobacteria*, and *Bacteroidetes*, while the control group was enriched with 12 biomarkers that belonged to *Firmicutes*. Welch's *t*-test was further employed to explore the differences in the microbial composition between the two groups (Figure 5). The results showed that the SC06 treatment significantly (*P* < 0.05) increased the abundances of *Bacteroidetes, Bacteroidales, Bacteroides, Fusobacteria, Clostridiaceae*, and *Veillonellaceae* in the cecum and simultaneously (*P* < 0.05) decreased the abundances of *Planococcaceae* in the duodenum, *Microbacteriaceae* in the jejunum, and *Lachnospiraceae, [Ruminococcus]* and *Ruminococcus* in cecum.

**Figure 6:**
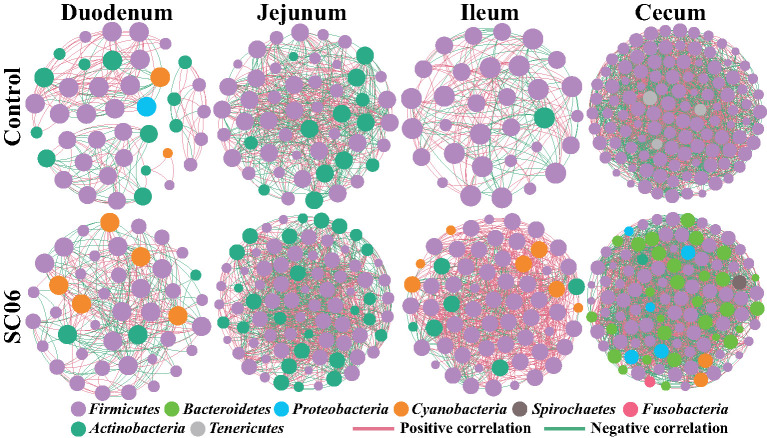
Co-occurrence networks of microbial communities based on Spearman correlation analysis sorted in color by operational taxonomic unit (OTU) level. A connection stands for a very strong (Spearman's R > 0.6) and significant [false discovery rate (FDR)-adjusted *P* < 0.05] correlation. The size of each node is proportional to the relative abundance; the thickness of each connection between two nodes is proportional to the value of Spearman's correlation coefficients. Red lines represent significant positive correlations, and green lines denote negative correlations.

To determine the co-occurrence patterns of intestinal microbiota in all groups, eight networks were constructed based on OTU levels (Figure 6, Table 6). The results showed that the microbial networks of SC06 group had more nodes (OTUs) and edges than those of the control group in the duodenum, jejunum, and ileum, except in the cecum. The values of average degree of SC06 group were higher than those of the control group in the duodenum, jejunum, ileum, and cecum. The values of graph density of SC06 group were lower than those of the control group in the duodenum and jejunum, whereas these were higher than those of the control group in the ileum and cecum. The modularity values of all the co-occurrence networks in the two groups were higher than 0.4. Additionally, a negative correlation of the network of the SC06 group was more than those of the control group in the jejunum and ileum, whereas it higher than that of the control group in the duodenum.

**Table 6:** Topological properties of co-occurrence network.

**Items**	**Duodenum**		**Jejunum**		**Ileum**		**Cecum**	
	**Control**	**SC06**	**Control**	**SC06**	**Control**	**SC06**	**Control**	**SC06**
Nodes	41	44	52	81	32	64	113	108
Edges	191	218	446	890	167	693	1704	1955
Average degree	9.317	9.909	17.154	21.975	10.438	21.656	30.159	36.204
Graph density	0.233	0.23	0.336	0.275	0.337	0.344	0.269	0.338
Modularity	0.639	0.542	0.423	0.493	0.495	0.523	0.573	0.422
Positive correlation	65.45%	49.54%	49.10%	54.27%	62.87%	79.22%	51.29%	51.56%
Negative correlation	34.55%	50.46%	50.90%	45.73%	37.13%	20.78%	48.71%	48.44%

## Discussion

Because of the issues of antibiotic-resistant bacteria and antibiotic-residual animal products, many countries have forbidden the use of antibiotics in animal husbandry (4). As an emerging green antibiotic substitute, probiotics are thought to be beneficial for the animals' health by maintaining the presence of beneficial microorganisms, enhancing digestive capacity, improving the mucosal immunity, and inhibiting pathogen adherence in the intestine (21, 22). Previous studies had reported that probiotics administration achieved a better effect on broilers' growth-related matrices than antibiotic administration (23–27). However, the beneficial effects of probiotics instead of antibiotics on broilers' growth performance are not always consistent, which are dependent on specific probiotic strains, the concentrations used, and the feeding stage of animals (28, 29). Many scientific literatures also documented that as an AGP alternative, probiotics had a similar effect on improving growth performance and gut health (8, 9, 21). In the current study, antibiotic-added basal diet was used as a control group to simulate the commercial intensive rearing mode, and a similar effect on the growth-related matrices of birds was observed in the two groups, which might imply that SC06 administration instead of antibiotics could bring similar economic benefits in the industrial farming of broilers.

Oxidative stress reflects an imbalance between the production of ROS (free radicals) and antioxidants, which leads to cellular dysfunctions and cell death (30). The major antioxidant defense system of the host is composed of antioxidant enzymes and biological antioxidants, of which T-AOC and SOD are the most important (31, 32). The elevated T-AOC capacity and SOD activity reflects the increase of antioxidant capacity (33). Liver redox environment is critical for the functions of the organ in nutrient digestion, so the redox status of the liver is vital for host health (34). In poultry husbandry, oxidative stress is ubiquitous, which restricts the growth performance of broilers (35, 36). There were several researches that demonstrated that probiotics could serve as a natural antioxidant to protect the host against oxidative stress-induced damage (37–39). Our results found that the T-AOC capacity and T-SOD activity in liver were significantly enhanced in the SC06 group, which is consistent with the previous findings that probiotics played a beneficial role in oxidative defenses (9, 40, 41).

Digestive enzymes play an important role in the digestion of nutrients into smaller nutrient molecules to facilitate the absorption by the host. Previous studies showed that probiotics had a positive effect in promoting intestinal digestive enzyme activities of broilers (42, 43). However, the positive results are not always consistent. Some literatures also found that probiotics did not affect the activities of intestinal digestive enzymes of broilers (44). The current study showed that probiotic SC06 significantly enhanced the activities of trypsin, AMS, and Na^+^K^+^-ATPase, while it markedly decreased lipase, γ-GT, and maltase activities in the ileum of broilers. These findings indicated that SC06 might play a biased beneficial role in degrading feed proteins and long-chain starch into smaller peptides, amino acids, short-chain dextrin, maltose, or glucose in order to be easily absorbed by the intestinal mucosa (45).

In the poultry industry, the immunosuppression induced by overcrowding, stress, and pathogenic infections can result in serious reductions in birds' growth performance, quality of poultry products, and economic benefits (46–48). Therefore, induction and maintenance of a proper level of immunologic function are vital for broiler healthy growth (23). Inflammatory cytokines secreted by the immune system play a key role in preventing against bacterial or viral infectious diseases and balancing the immune homeostasis (49). The pro-inflammatory cytokines (IL-6, TNF-α) and anti-inflammatory cytokines (IL-10, TGF-β) are key inflammatory cytokines that endorse cell-mediated immunity and regulate cytokine secretion homeostasis (50, 51). Many documents reported that probiotics could modulate host immune function (52, 53) and have been widely used in animal and human in order to enhance the disease resistance capacity (54, 55). As a natural immune modulator, probiotics could improve the immune response of broilers by increasing inflammatory cytokines and thus protect broilers against pathogens, coccidia, viruses, and stress (56, 57). In the present study, probiotic SC06 significantly increased the anti-inflammatory cytokine (IL-10) and decreased the pro-inflammatory cytokines (IL-6 and TNF-α) in the ileum, which implies that probiotic SC06 could be used as a potential immune modulator to regulate the intestinal immune function of broilers.

One of the most important functions of the intestinal mucosa is to act as an intestinal epithelial barrier that consists of the tight junction, adhesion belt, and desmosomes (58). The intestinal epithelial barriers play a vital role in resisting invasion of pathogens and the maintenance of mucosal homeostasis (59, 60). Many studies have reported that supplementation with probiotics improved the intestinal ultrastructure of broilers (35, 61). Similarly, we also found that SC06 treatment enhanced the intercellular connectivity in ileal mucosa, as evidenced by longer tight junctions and adhesion belts, indicating that SC06 could improve the intestinal epithelial barriers of broilers.

Gut microbiota plays an important role in the digestive tract of animals (62). The composition of the intestinal microbial community is greatly influenced by dietary interaction (63). Previous studies have demonstrated that dietary probiotics have a positive effect in modulating the intestinal microbiota (21, 23, 64). Supplementation with probiotics could promote the presence of beneficial bacteria and reduce potential harmful bacteria populations in the intestinal tract of chickens (65, 66) and reestablish pathogen-induced intestinal microbial dysfunction (67). In the present study, dietary SC06 mainly increased the alpha diversity indices in the jejunum, ileum, and cecum, indicating that SC06 improved the microbial diversity of the above intestinal segments. LefSe results showed that SC06 induced differentially enriched bacterial species at different taxonomic levels, especially in the cecum. The SC06 group was totally enriched with 51 biomarkers (microbial taxa) in the duodenum, jejunum, and cecum, most of which belonged to *Firmicutes, Proteobacteria*, and *Bacteroidetes*. We further found that among the identified 68 biomarkers (microbial taxa) in the two groups, 11 biomarkers were calculated to be significantly different between the two groups by Welch's *t*-test analysis. Dietary SC06 significantly increased the abundances of *Bacteroidetes, Bacteroidales, Bacteroides, Fusobacteria, Clostridiaceae*, and *Veillonellaceae* in the cecum. *Bacteroides* genus can metabolize a variety of plant- and animal-derived glycans and improve the immune function and mucosal barrier function of animals (68, 69). It is reported that *Fusobacteria* activate the inflammatory responses of the host to protect against pathogens that promote tumor growth (70). *Clostridiaceae* mediates starch breakdown and lactic acid fermentation (71) and is one of the three key families in dogs to digest the intestinal protein and energy (72). *Veillonellaceae* was reported to produce high levels of the short-chain fatty acids (acetate and propionate) (73). Simultaneously, SC06 treatment decreased the abundances of *Planococcaceae* in the duodenum, *Microbacteriaceae* in the jejunum, and *Lachnospiraceae* and *Ruminococcus* in the cecum. *Planococcaceae* family previously was described in association with vertebrate carrion (74). *Lachnospiraceae* was reported to increase in patients with non-alcoholic fatty liver disease (75). It was reported that antibiotic (zinc bacitracin) treatment increased the relative abundance of *Ruminococcus*, but the role of this genus in broilers remains to be further investigated (76, 77). The above results implied that SC06 supplementation may exert beneficial effects on modulating the intestinal microbiota of broilers.

Finally, the co-occurrence network analysis was employed to investigate microbial interactions. In this study, we found that the values of edges and average degree of the microbial networks in the SC06 group were higher than those in the control group in the different intestinal segments, suggesting that SC06 treatment increased the connection among the intestinal microbiota (78). The modularity values of all the co-occurrence networks in the two groups in different intestinal segments were higher than 0.4, suggesting that these microbial networks had a modular structure (79). Additionally, negative connection of the network in the SC06 group was less than that in the control group in the jejunum and ileum, which could be interpreted as a reduction in competitive relationships within the intestinal microbiota (80).

## Conclusion

In conclusion, *B. amyloliquefaciens* SC06 instead of antibiotics is beneficial for the health of broilers by improving the antioxidant capacity of the liver, digestive function and immune response of the intestinal mucosa, and intestinal epithelial barrier and modulating the intestinal microbiota. However, further investigations about the involved and interacted roles of gut microbiota in SC06-mediated benefits are needed.

## Data Availability Statement

The datasets presented in this study can be found in online repositories. The names of the repository/repositories and accession number(s) can be found at: NCBI SRA BioProject, accession no: PRJNA720313.

## Ethics Statement

The animal study was reviewed and approved by the Institutional Animal Care and Use Committee of Zhejiang University, P. R. China.

## Author Contributions

W-FL contributed to the conceptualization, supervision, and project administration. BW and YZ contributed to the microbial analysis, writing of the original draft, and writing, review, and editing. BW, YZ, and LT contributed to the animal experiments. ZZ, LG, and YW assisted with the experiments and manuscript preparation. All authors have read and agreed to the published version of the manuscript.

## Conflict of Interest

The authors declare that the research was conducted in the absence of any commercial or financial relationships that could be construed as a potential conflict of interest.
